# Multi-Omics Analysis Reveals the Regulatory Mechanism of Different Probiotics on Growth Performance and Intestinal Health of *Salmo trutta* (*S. trutta*)

**DOI:** 10.3390/microorganisms12071410

**Published:** 2024-07-12

**Authors:** Mengjuan Chen, Zhitong Wang, Hui He, Wenjia He, Zihao Zhang, Shuaijie Sun, Wanliang Wang

**Affiliations:** 1 Institute of Fisheries Science, Tibet Academy of Agriculture and Animal Husbandry Sciences, Lhasa 850032, China; chen_333666979@163.com (M.C.); 18963717463@163.com (Z.W.); h18375399868@163.com (H.H.); xzshuichansuo@163.com (W.H.); zhangzh180616@163.com (Z.Z.); 15738816518@163.com (S.S.); 2 College of Animal Science and Technology, Henan Agricultural University, Zhengzhou 450046, China; 3 Indigenous Fish Breeding and Utilization Engineering Research Center of Xizang, Lhasa 850032, China; 4 Key Laboratory of Fishery and Germplasm Resources Utilization of Xizang Autonomous Region, Lhasa 850032, China

**Keywords:** *Salmo trutta*, probiotics, gut microbiome, gut metabolome, intestinal function

## Abstract

Probiotics play an important role in animal production, providing health benefits to the host by improving intestinal microbial balance. In this study, we added three different probiotics, *Saccharomyces cerevisiae* (SC), *Bacillus licheniformis* (BL), and *lactic acid bacteria* (LAB), and compared them with the control group (CON), to investigate the effects of probiotic supplementation on growth performance, gut microbiology, and gut flora of *S. trutta*. Our results showed that feeding probiotics improved the survival, growth, development, and fattening of *S. trutta*. Additionally, probiotic treatment causes changes in the gut probiotic community, and the gut flora microorganisms that cause significant changes vary among the probiotic treatments. However, in all three groups, the abundance of *Pseudomonas*, *Acinetobacter*, and *Rhizophagus* bacterial genera was similar to that in the top three comparative controls. Furthermore, differences in the composition of intestinal microbiota among feed types were directly associated with significant changes in the metabolomic landscape, including lipids and lipid-like molecules, organic acids and derivatives, and organoheterocyclic compounds. The probiotic treatment altered the gut microbiome, gut metabolome, and growth performance of *S*. *trutta*. Using a multi-omics approach, we discovered that the addition of probiotics altered the composition of gut microbiota, potentially leading to modifications in gut function and host phenotype. Overall, our results highlight the importance of probiotics as a key factor in animal health and productivity, enabling us to better evaluate the functional potential of probiotics.

## 1. Introduction

*Salmo trutta fario Linnaeus* belongs to *Salmonidae*, *Salmoninae*, *Salmo*. It is native to Europe, northern Africa, and western Asia and was introduced into the Yadong area of Tibet in the late 19th century. Following long-term adaptation, Salmo trutta fario Linnaeus has successfully reproduced and thrived in its natural environment, maintaining a stable population. This species holds significant economic value in Tibet and is classified as a second-class protected animal in the Tibet Autonomous Region [1,2]. In recent years, the widespread adoption of standardized intensive breeding practices has led to the extensive use of antibiotics for disease prevention and treatment. However, this has resulted in significant antibiotic residues and the proliferation of antibiotic-resistant bacteria, posing challenges for effectively managing diseases in breeding production. As a result, the intestinal flora of fish is disrupted and their immune system is compromised. Additionally, the presence of residual antibiotics in aquatic products may constitute a potential risk to human health [3,4]. To address the challenges related to aquaculture diseases and food safety, and to ensure the sustainable and healthy development of aquaculture, there is a growing focus on researching environmentally friendly, safe, and effective alternatives to antibiotics.

The word probiotics, which is derived from the Greek word “pro bios” that means “for life” [5], refers to a group of organisms that are a normal part of the intestinal tract and can create compounds with positive effects. Probiotics, as microbial feed additives, confer health benefits to the host by modulating intestinal microbiota. They are frequently used in aquaculture because of their capacity to synthesize a variety of extracellular enzymes that have a positive impact on host immunology, growth performance, and feed conversion [6]. Additionally, many probiotics provide vitamins, fatty acids, and essential amino acids to the host [7,8,9]. Several studies have shown that probiotics, alone or in combination, can enhance local or systemic immunity in fish [10,11,12]. Microorganisms commonly used as probiotics in aquaculture include yeast, bacteria, and algae [13]. Kosaza evaluated whether boosting the diet with *Bacillus* toyoda could accelerate the growth rate of yellowtail for the first time in aquaculture [14]. The findings of Vazirzadeh et al. demonstrated that the addition of Saccharomyces cerevisiae to the feed of rainbow trout improved immunity and FCR, providing a significant positive effect on immunomodulation, as well as resistance to pathogenic bacterial infections [15]. These findings suggest that probiotics are promising natural products for increasing the sustainability and efficiency of aquaculture.

Therefore, this study employed metagenomics to investigate the impact of different probiotics (*Saccharomyces cerevisiae* (SC), *Bacillus licheniformis* (BL), and *lactic acid bacteria* (LAB)) on the functional diversity of intestinal flora. Concurrently, non-targeted metabolomics was utilized to analyze changes in intestinal microbial metabolites following the addition of these probiotics, enhancing our understanding of previously unknown microbial metabolites. Ultimately, through integrated multi-omics analysis, this study offers new insights into the role of different probiotics in aquaculture and sheds light on the mechanisms by which probiotics promote the growth, development, and disease resistance of *Salvelinus* asiaticus.

## 2. Material and Methods

### 2.1. Probiotics Source

#### *S. trutta* and Trial Design

*S. trutta* were sourced from the Yarlung Zangbo River Fishery Resources Breeding Base in Lhasa, Tibet Autonomous Region. Prior to experimental feeding, 360 healthy *S. trutta* with an average weight of 84.57 ± 1.83 g were selected and raised in a cement pond (10 m × 2.0 m × 1.2 m) for 10 days. Proprietary feed formulations were selected (Tables S1 and S2). Then, after 24 h of starvation, the salmon were randomly assigned to 12 cages (2.0 m × 1.0 m × 1.2 m), the water level was controlled by 0.6 m, and 30 *S. trutta* were divided into four groups and placed into each cage, with three replicates in each group. The control group was fed basic feed, and SC, BL, and LAB probiotics were added to the treatment group. Probiotics were purchased from Cangzhou Yihong Biotechnology Co., Ltd., Cangzhou, China, and the effective viable count was 1.0 × 10^7^ CFU/g. The feed after adding probiotics was stored in a refrigerator at 4 °C. Feeding twice daily, with each feeding comprising 3% of their body weight, was implemented. The water temperature was maintained at 10~12 °C, and dissolved oxygen of 6.2~7.3 mg/L. The main components of LAB were *Lactobacillus acidophilus* and *Lactobacillus plantarum*. An experimental feeding trial was carried out over a 3-month period. In addition, the unfed residual bait was collected in time after feeding every day, and the actual feed intake was calculated according to the initial feeding amount after drying.

### 2.2. Feed and Sample Collection

At the end of animal study, after 24 h of fasting, all *S. trutta* were euthanized by administered intramuscularly with Tricaine methanesulfonate (MS-222) (MCE Co., Ltd., Malta, NJ, USA) at a concentration of 130 mg/L. Five tails were randomly selected in each cage, and 50 mg of intestinal contents were sampled from the same region of the distal intestine. Samples were immediately frozen on dry ice and subsequently transferred to a −80 °C freezer within hours. Each sample was kept on dry ice during transportation.

### 2.3. Growth Performance Analysis

The initial weight, length, fatness, and liver index of each fish were measured, and the number of surviving fish in the different probiotic supplementation groups was calculated. WG (final weight in g − initial weight in g), SGR ((ln (final weight in g) − ln (initial weight in g) × 100)/t (days)), hepatic steatosis index (HSI; liver weight in g/body weight in g) × 100), and condition factor (K = (W/L^3^) × 100) were recorded [16].

### 2.4. Metagenomic Analysis

#### 2.4.1. Genomic DNA Extraction, Library Preparation, and Sequencing

Genomic DNA extraction and shotgun metagenomic sequencing were carried out at Biomarker Technologie Co., Ltd. (Beijing, China). Total microbial genomic DNA was extracted from the gut using the CTAB method. The NanoDrop ND-1000 spectrophotometer (Invitrogen, Qubit3.0, Waltham, MA, USA) and agarose gel electrophoresis were used to evaluate the quality and quantity of the extracted DNA. The qualified DNA was used to construct the shotgun metagenomic sequencing library with VAHTS^®^ Universal Plus DNA Library Pren Kit (Illumina, San Diego, CA, USA). The library passed the QSEP-400 inspection for quality and was quantified using Qubit 3.0 for the library concentration. Illumina Novaseq (Illumina, San Diego, CA, USA) was used as the sequencing platform. The cluster density was within 1.255–1.412 K cluster/mm^2^ range, and the sequencing operational error rate was <0.05.

#### 2.4.2. Metagenomic De Novo Assembly, Gene Prediction, and Annotation

Clean reads were obtained by filtering the original sequence obtained by sequencing, and MEGAHIT v0.1-beta [17] was used to assemble the macroscopic group. Quast 5.0.0 [18] software evaluated the assembly results. The encoding area in the genome was identified using agnemark (http://exon.gatech.edu/GeneMark/metagenome, 5 March 2023) [19] software by default parameters (-A-D-F-G). To obtain a non-redundant gene catalog, redundancy was removed using MMseq2v2 software with the similarity threshold set at 95 and the threshold set to 90. Protein sequences of non-redundant genes and the Nr database [20] were used to align with BLAST to find the most similar sequences in the Nr database and annotate. Protein sequences of non-redundant genes and protein sequences included in the KEGG database were used for BLAST alignment (diamond v0.9.29 alignment screening threshold E-value 1 × 10^−5^), and functional genes were annotated. The beta diversity of microbial composition was assessed using Bray–Curtis distance metrics and displayed through principal coordinate analysis (PCoA) and non-metric multidimensional scaling (NMDS) hierarchical clustering [21,22]. According to the abundance and variation of each species in each sample, the correlation analysis (including positive and negative correlation) was performed by using the Spearman algorithm, and statistical tests were performed to filter the data groups with a correlation greater than 0.5, and *p*-values less than 0.05, and correlation network diagrams were plotted. Use Python 3.11.2 to analyze and visualize data.

### 2.5. Metabolomics Analysis

#### 2.5.1. Metabolite Extraction and Treatment

Intestinal samples were weighed 50 mg, ground in a 2 mL Eppendorf tube containing steel beads, added 1 mL precooled mixtures of methanol, acetonitrile and water (*v*/*v*/*v*, 2:2:1) for 10 min at 45 Hz in a grinding mill and then placed for 1 h into ice baths with ultrasonic shaking. Subsequently, the mixture was placed at −20 °C for 1 h and centrifuged at 4 °C and 12,000 rpm for 15 min. The 500 μL supernatant was taken, dried and redissolved using a mixture of 160 μL acetonitrile and water (volume ratio: 1:1), vortexed for 30 s, ultrasonic in ice baths for 10 min and then centrifuged at 4 °C, 12,000 rpm for 15 min. Additionally, quality control (QC) samples are normalized by pooling aliquots from all representative samples and used for data normalization.

#### 2.5.2. LC-MS/MS Analysis

Waters Xevo G2-XS QTOF (Waters Co. Ltd., Milford, MA, USA) high-resolution mass spectrometer can collect primary and secondary mass spectrometry data in MSe mode under the control of the acquisition software (MassLynx V4.2, Waters). In each data acquisition cycle, dual-channel data acquisition can be performed on both low collision energy and high collision energy at the same time. The low collision energy is 2 V, the high collision energy range is 10~40 V, and the scanning frequency is 0.2 s for a mass spectrum. The parameters of the ESI ion source are as follows: capillary voltage, 2000 V (positive ion mode) or −1500 V (negative ion mode); cone voltage, 30 V; ion source temperature, 150 °C; desolvent gas temperature, 500 °C; backflush gas flow rate, 50 L/h; desolventizing gas flow rate, 800 L/h.

#### 2.5.3. Data Preprocessing and Annotation

The raw data collected using MassLynx V4.2 was processed by Progenesis QI v3.0 software for peak extraction, peak alignment, and other data processing operations, based on the Progenesis QI software online METLIN database and Biomark’s self-built library for identification, and at the same time, theoretical fragment identification and mass deviation were all within 100 ppm.

#### 2.5.4. Data Analysis

After normalizing the original peak area information with the total peak area, follow-up analysis was performed. Principal component analysis and Spearman correlation analysis were used to determine the reproducibility of the group samples and quantitative control samples. The identified compounds were categorized and pathway information was retrieved in KEGG, HMDB, and lipidmaps databases, the multiplicity of differences was calculated and compared based on the grouping information, and the *t*-test was used to calculate the significance of difference *p* value of each compound. OPLS-DA modeling was performed using the R language package ropls, and 200 permutation tests were performed to verify the reliability of the model. The VIP value of the model was calculated using multiple cross-validation. The method of combining the difference multiple, the *p*-values and the VIP value of the OPLS-DA model was adopted to screen the differential metabolites. The screening criteria were fold change (FC) >1, *p*-value, and VIP > 1. The differential metabolites of KEGG pathway enrichment significance were calculated using hypergeometric distribution test.

### 2.6. Correlation Analysis between Microbiome and Metabolome

Correlation analysis between metabolome and microbiome was calculated by Top20 differential metabolites with the lowest *p*-values. Bacteria as Top20 abundance microorganisms under genus level which accounted for more than 80% of the microbial sequence reads and metabolites were deduced from the differential KEGG metabolic pathways.

## 3. Results

### 3.1. Survival and Growth Performance Analysis

The survival rate, growth parameters, fatness, and liver index of *S. trutta* reared with each probiotic are listed in Table 1. Our results showed that the addition of probiotics improved the survival rate of *S. trutta* (*p* < 0.05). There were no significant differences among the liver body indices of BL, SC, LAB, and CON groups. Compared to the control group, the probiotic-supplemented group showed a significant increase in body weight and length (*p* < 0.05). Additionally, only the BL group showed a significant increase in probiotic-supplemented *S. trutta* fat compared to the control group (*p* < 0.05).

### 3.2. Effect of Probiotics Addition on Intestinal Microbial Composition and Function in S. trutta

**Figure 1:**
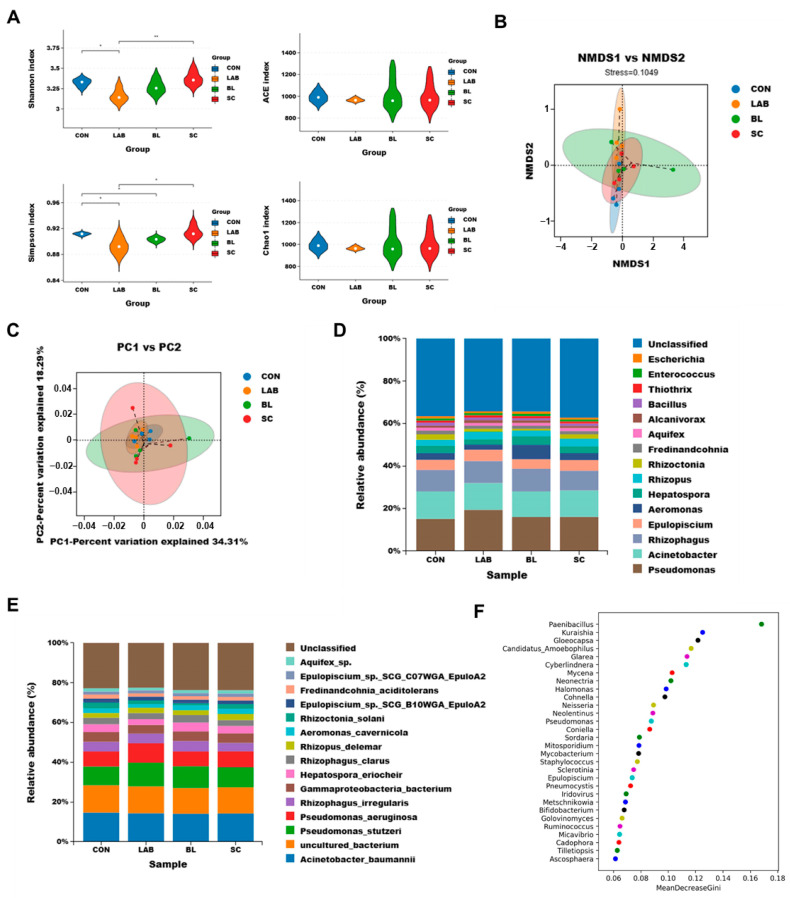
Map of macrogenomic analysis of the gut of *S. trutta* between control and probiotic groups. (**A**) Gut bacterial alpha diversity, including Shannon index, ACE index, Simpson index, and Chao1 index. * *p* < 0.01, ** *p*  < 0.01. (**B**) NMDS based on Bray–Curtis distance. (**C**) PCoA based on Bray–Curtis distance. (**D**) Differences in microbial community composition at the genus level. (**E**) Differences in microbial community composition at the species level. (**F**) The top 30 most important bacterial communities identified by random forest classification in control and probiotic groups. BL: *Bacillus licheniformis*, SC: *Saccharomyces cerevisiae*, LAB: *lactic acid bacteria*.

To achieve a comprehensive comparison, we used metagenomics to detect the gut microbial composition, diversity, richness, and functional enrichment. First of all, taxonomic alpha diversity was estimated using the Shannon index, ACE index, Simpson index, and Chao1 index (Figure 1A). The NMDS analysis, based on β diversity, showed variations in the distances among samples’ passing points. The lower the stress value, the better. The CON, SC, BL, and LAB samples demonstrated some overlap (Figure 1B), indicating that there might be some difference among the gut microbiota components in each group. Then, using principal component analysis (PCA) to conveniently observe the variation law between samples, the results showed significant separation and some degree of clustering of bacterial community composition among the four groups (Figure 1C).

Then, to further observe the effect of probiotic addition on the gut microbiota of *S. trutta*, we investigated the microbial species and their relative abundance at the genus and species levels. As shown in Figure 1D,E, the community structure and abundance of all bacteria in these groups were very similar and mainly comprised of Acinetobacter and Pseudomonas. Compared with CON, the abundance of *Hepatospora*, *Aeromonas*, and *Rhizoctonia* were decreased and the abundance of *Pseudomonas*, *Rhizopus*, and *Alcanivorax* were increased in LAB. The abundance of *Rhizoctonia* genus was decreased and *Aeromonas*, *Hepatospora*, and *Alcanivorax* were increased in BL. The abundance of *Rhizoctonia* was decreased and *Rhizopus* was increased in SC. At the species level, consistent increases or decreases were observed in all samples at the genus level (Figure 1E). Random forest analysis can identify key species that differ between samples. The random forest analysis (Figure 1F) showed that the key species with the top five differences were *Paenibacillus*, *Kuraishia*, *Gloeocapsa*, *Candidatus_Amoebophilus*, and *Glarea*.

### 3.3. Functional Prediction of Microbe Community

**Figure 2:**
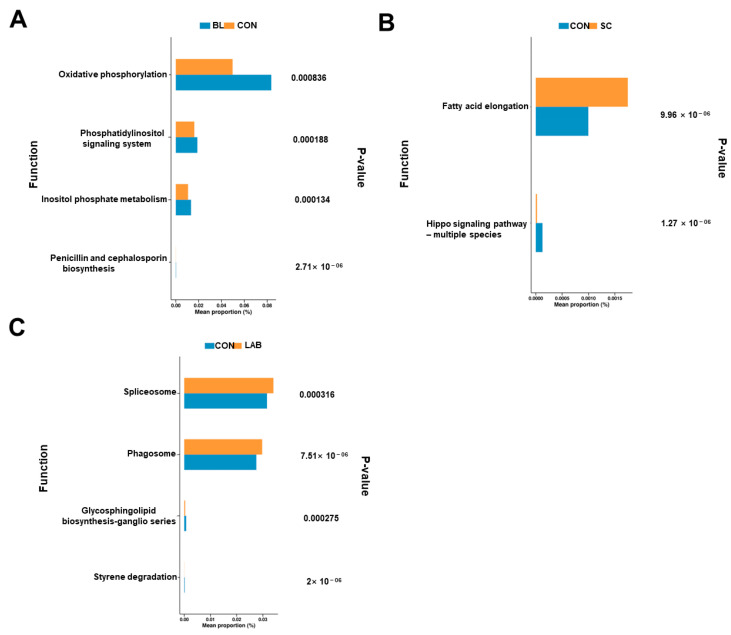
Functional enrichment analysis of differential metabolic flora. (**A**) Histogram of functional enrichment analysis of differential flora in CON and BL groups. (**B**) Histogram of functional enrichment analysis of differential flora in CON and SC groups. (**C**) Histogram of functional enrichment analysis of differential flora in CON and LAB groups. BL: *Bacillus licheniformis*, SC: *Saccharomyces cerevisiae*, LAB: *lactic acid bacteria*.

To further understand the functional differences between CON and microorganisms, KEGG analysis of differentially functional genes was performed according to the criterion of nonparametric testing (*p* < 0.05). The results showed that “oxidative phosphorylation, phosphatidylinositol signaling, inositol phosphate metabolism, and penicillin and cephalosporin biosynthesis” pathways decreased after the addition of BL probiotics (Figure 2A). In addition, we were able to learn that the production of fatty acids, as well as abundance of the hippo signaling pathway, were altered after the addition of probiotics (Figure 2B). Additionally, compared with CON, increased enrichment was observed in “Glycosphingolipid biosynthesis ganglio series and Styrene degradation” and decreased enrichment was observed in “Spliceosome and Phagosome” after LAB addition (Figure 2C). 

### 3.4. Quality Control

**Figure 3:**
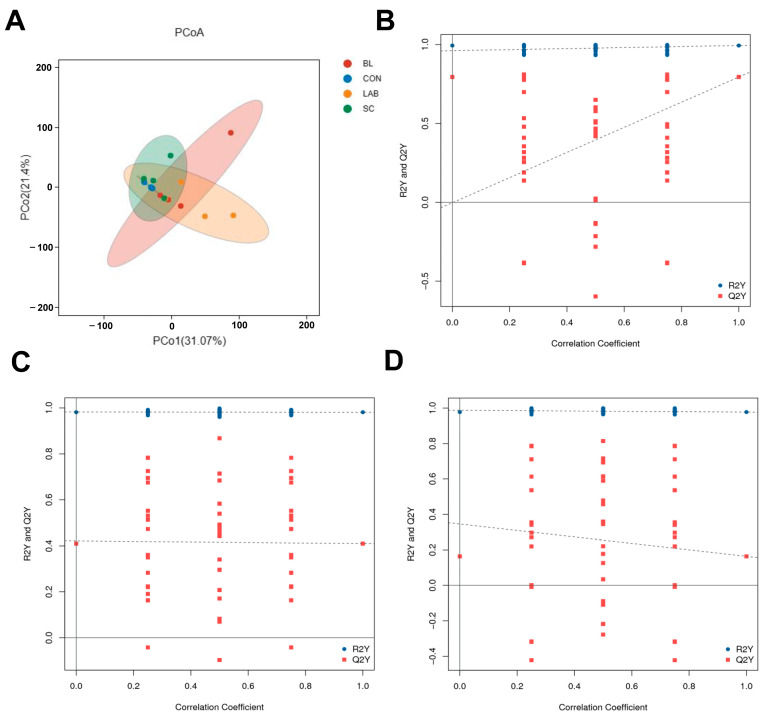
Quality control of the metabolomics data. (**A**) Principal coordinates analysis (PCoA) plots between the control and probiotics groups. (**B**) Orthogonal partial least squares discriminant analysis (OPLS-DA) score plot between CON group and SC group. (**C**) Orthogonal partial least squares discriminant analysis (OPLS-DA) score plot between CON group and BL group. (**D**) Orthogonal partial least squares discriminant analysis (OPLS-DA) score plot between CON group and LAB group. The blue and red dots represent the R_2_Y and Q_2_Y of the post-replacement model, respectively, and the two dashed lines are regression lines fitted to R_2_Y and Q_2_Y. BL: *Bacillus licheniformis*, SC: *Saccharomyces cerevisiae*, LAB: *lactic acid bacteria*.

PCA is an unsupervised pattern recognition method for statistical analysis of multidimensional data that converts a set of potentially correlated variables into a set of linearly uncorrelated variables through orthogonal transformation. The PCA of samples provided a preliminary understanding of the overall metabolic differences between groups and the magnitude of variability between samples within groups. As shown in Figure 3A, the PCoA results indicated that the CON group was significantly separated from the BL and LAB groups, whereas it was not significantly separated from the SC group, and the sample distribution of the CON group converged. The OPLS-DA model is a latent variable regression method based on the covariance between predictor variables and the response. The prediction parameters of the evaluation model were R_2_X, R_2_Y, and Q_2_Y, where R_2_X and R_2_Y represent the interpretation rate of the established model to X and Y matrices, respectively, and Q_2_Y represents the predictive ability of the model. The closer that R_2_Y and Q_2_Y of the index are to 1, the more stable and reliable the model is, and the differential metabolites can be screened by this model. The R_2_Y and Q_2_Y values of OPLS-DA were 0.998 and 0.441, respectively, in the BL group compared to those in the control group (Figure 3B). The R_2_Y and Q_2_Y values of OPLS-DA were 0.981 and 0.41, respectively, in the SC group compared with those in the control group (Figure 3C). Compared to the control group, the R_2_Y and Q_2_Y values of OPLS-DA in the LAB group were 0.977 and 0.164, respectively (Figure 3D).

### 3.5. Analysis of Differential Metabolites and Their KEGG Enrichment in Control and Probiotic-Treated Groups

**Figure 4:**
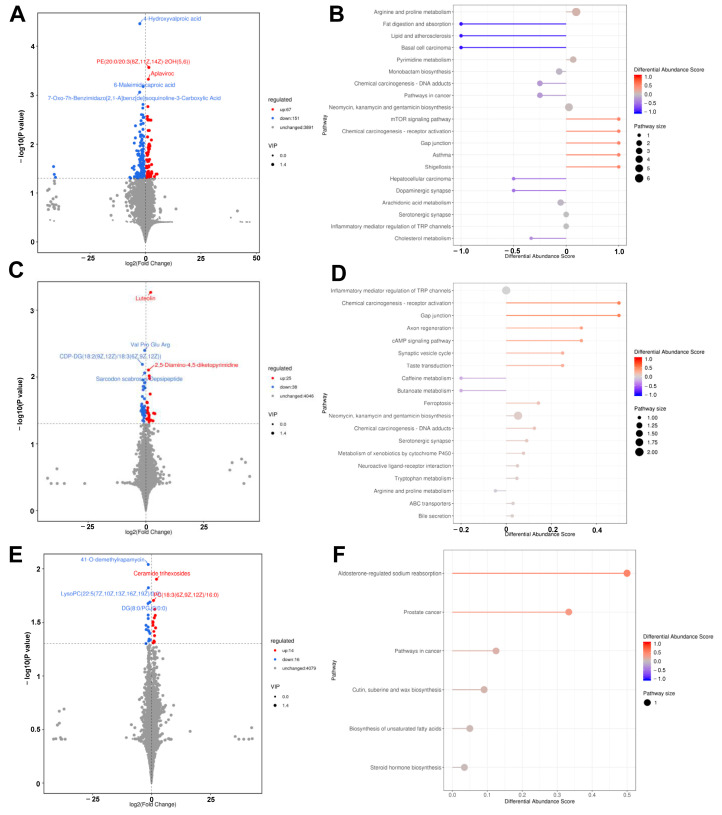
Different metabolites and metabolic pathways between the control group and the probiotics-treated group. Differential metabolite volcano plots of the BL (**A**), SC (**C**), and LAB (**E**) groups, respectively, versus the CON group. Each point represents a metabolite, the points highlighted in blue are downgrade metabolites while those in red are upgrade metabolites, and the size of the point represents the variable importance in projection. Differential metabolic pathways of the BL (**B**), SC (**D**), and LAB (**F**) groups, respectively, versus the CON group. The length of the line segment indicates the absolute value of DA score, and the size of the dots at the endpoints of the line segment indicates the number of differential metabolites in the pathway. The color of the line segments and dots reflects the size of the *p*-value, with a more red color indicating a smaller *p*-value and a more blue color indicating a larger *p*-value. BL: *Bacillus licheniformis*, SC: *Saccharomyces cerevisiae*, LAB: *lactic acid bacteria*.

At FC = 1 and *p*-value = 0.05, there were 199 differential metabolites in the BL group compared to the control group, of which 102 metabolites were upregulated and 97 were downregulated (Figure 4A). Compared to the control group, there were 63 differential metabolites in the SC group, of which 25 were upregulated and 38 were downregulated (Figure 4C). There were 30 differential metabolites in the LAB group compared to those in the control group, of which 14 were upregulated and 16 were downregulated (Figure 4E).

**Figure 5:**
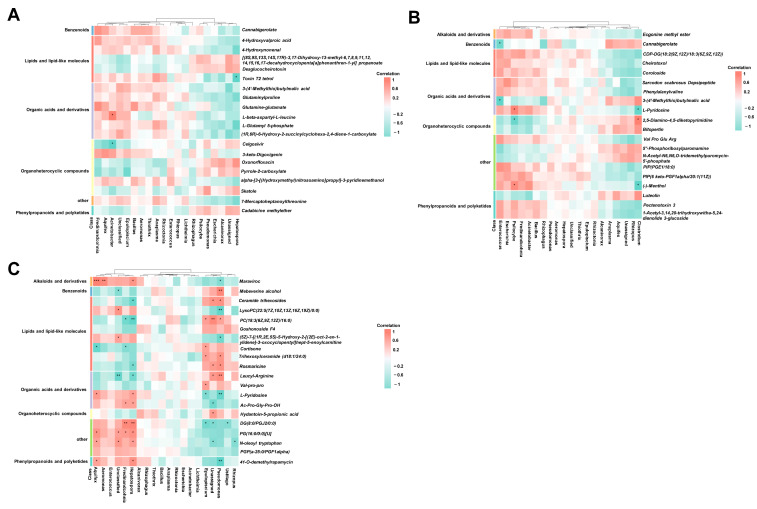
Spearman’s correlation analysis of microbiome and metabolism. Correlation analyses were performed between the control group and the BL group (**A**), SC group (**B**) and LAB group (**C**), respectively. Bacteria were deduced from the top 20 genera, which accounted for more than 80% of the microbial sequence reads, and metabolites were deduced from the different metabolic pathways by stages. The color represents the correlation coefficient. Only correlation coefficient beyond 0.6 and *p*-value below 0.05 were considered significant; in figures, ***, ** and * denote, respectively, *p*-values below 0.001, 0.01 and 0.05. BL: *Bacillus licheniformis*, SC: *Saccharomyces cerevisiae*, LAB: *lactic acid bacteria*.

Complex metabolic reactions and their regulation in organisms are not performed alone and are often performed by different genes and proteins to form complex pathways and networks, and their mutual influence and regulation eventually lead to systemic changes in the metabolome. The KEGG database was used to study genes, expression information, and metabolite content in the overall network. The results are shown in Figure 5. Metabolism of arginine and proline; digestion and absorption of fat, lipids, and atherosclerosis; biosynthesis of neomycin, kanamycin, and gentamicin; and the mTOR signaling pathway were enriched after the addition of BL probiotics (Figure 4B). After adding SC probiotics, the inflammatory mediator regulation of TRP channels, CAMP signaling pathway, and neomycin, kanamycin, and gentamicin biosynthesis signal pathways were enriched. Additionally, some amino acid metabolic pathways were enriched (Figure 4D). In the LAB group, the biosynthesis of unsaturated fatty acids; aldosterone-regulated sodium reabsorption; and cutin, suberin, and wax biosynthesis signal pathways were enriched (Figure 4F). Overall, after the addition of probiotics, pathways related to inflammation, amino acid metabolism, and fatty acid metabolism were enriched, indicating that probiotics play a role in intestinal inflammation and fat metabolism.

### 3.6. Correlation Analysis between Microbiome and Metabolism

To investigate the association between specific metabolites and the presence of microbes, we verified the correlation between the intestinal microbiota and metabolites in the control group and groups treated with different probiotics. Correlation analysis was performed by calculating the Spearman’s correlation coefficients and *p*-values. Otherwise, metabolites from different metabolic pathways were selected. As shown in Figure 5A, in the BL treatment group compared to the control group, *Hepatospora* was negatively correlated with Toxin T2 tetrol, 4-Hydroxynonenal, Glutaminylproline, and L-glutamyl 5-phosphate, and *Acinetobacter* was positively correlated with L-beta-aspartyl-L-leucine and negatively correlated with Celgosivi. Finally, the classification showed that most differential metabolites included lipids, lipid-like molecules, organic acids and derivatives, and organoheterocyclic compounds. When the SC-treated group was compared to the control group, the results showed that *Enterococcus* was negatively correlated with cannabigerolate and 3-(4’-Methylthio) butylmalic acid, and *Psilocybe* was positively correlated with L-Pyridosine (Figure 5B). Similar to the BL group, the correlated metabolites in the SC group were mainly clustered as lipids and lipid-like molecules, organic acids and derivatives, and organoheterocyclic compounds. We speculated that organoheterocyclic compounds may be related to the production of antimicrobial substances in the gut that counteract bacterial cell walls.

In the LAB-treated group, *Aquifex* showed a positive correlation with Maraviroc, L-pyridosine, PG(16:0/0:0)[U], N-oleoyl tryptophan, and 41-O-demethylrapamycin and a negative correlation with cortisone (Figure 5C). Many microorganisms and their metabolites were negatively and positively correlated. These metabolites were classified into lipids, benzenes, and amino acids. Interestingly, this result was consistent with the pathways enriched by differential metabolites, suggesting that lipid anabolism in the gut of Adonis salmon changed mainly after the addition of probiotics. Furthermore, most of these metabolites are benzene molecules, which may be antimicrobial substances produced in the intestine, thus boosting intestinal immunity.

## 4. Discussion

In this study, we investigated the effects of probiotic supplementation on the intestinal microbiota and metabolomes of fish. Our results revealed noteworthy differences in the intestinal function, gut microbiota composition, and metabolite profiles between the control and probiotic-supplemented groups. Correlation analysis further confirmed significant associations between the microbial community and a multitude of metabolites. To elucidate these findings, we employed advanced techniques such as deep sequencing-based metagenomics and untargeted metabolomics, enabling us to visually illustrate alterations in both the microbial community and metabolite profiles. Collectively, our findings shed light on the intricate relationship between probiotic supplementation and distinctive functional and metabolic characteristics of the gut microbiota in *S. trutta*.

Fish are susceptible to different types of bacterial, fungal, and parasitic infections during their growth and development, which are threats to fish health. Probiotics have been shown to be positive promoters of growth, survival, and health in aquatic animals [10]. As natural products, probiotics have a great potential to improve the efficiency and sustainability of aquaculture production [6]. *Saccharomyces cerevisiae* is one of the most popular probiotics in extract or whole cell form, and it is considered an important source of products with probiotic activity [23]. Many studies have shown that it can promote fish growth, development, and feed utilization and improve intestinal health and intestinal mucosal flora [24,25,26,27,28]. The results of this study showed that the body weight and length of *S. trutta* significantly increased after the addition of SC, indicating that SC could promote the growth and development of *S. trutta*. As the most commonly used beneficial probiotics in animal diets, lactic acid bacteria significantly contribute to maintaining the intestinal ecosystem and stimulating the host immune system [29,30]. *Bacillus licheniformis* is also widely added to aquaculture feeds because of its resistance to harsh environments and ability to produce various enzymes [31]. Some researchers have demonstrated that the growth of grass carp improved after supplementation with *Bacillus Licheniformis* [32], which is consistent with the results of this study. These findings suggest that probiotics promote the growth and development of *S. trutta*. Therefore, probiotics as feed additives can also provide economic benefits for industrial farming of fisheries.

Notably, the addition of probiotics altered the gut microbial composition and diversity in *S. trutta*, and the three most abundant groups were *Pseudomonas*, *Acinetobacter*, and *Rhizophagus*. Compared to the control group, the SC group *Rhizophagus_irregulari* and *Rhizoctonia_solani* decreased. *Rhizophagus rickettsii* is an aggressive, soil-borne, semi-viviparous trophic pathogen that can destroy many plants [33]. However, no such studies have been conducted on fish. We speculated that it may also be harmful to fish. Notably, the level of *Hepatospora* abundance decreased after the addition of LAB. *Hepatospora* are intracellular parasites that infect epithelial cells of the liver, bile, and pancreas in animals [34]. Ding performed transcriptome sequencing before and after infection and found that the differentially expressed genes were mainly enriched in amino acid metabolism and fatty acid biosynthesis [35]. This result is consistent with the pathways enriched by differential metabolites identified in the metabolome sequencing results of this experiment. The results for the gut microbes were consistent with those of other studies, suggesting that probiotics have a positive effect on gut health.

Analysis of macrogenomic results has provided a clear understanding of the changes in the intestinal flora following probiotic addition; however, these changes in the intestinal flora lead to changes in certain metabolites that we do not understand. Studies have also highlighted that the metabolites of gut microbes, such as bile acids, short-chain fatty acids, and amino acids, play a particularly important role in gut health [36]. Therefore, we next performed metabolomics to gain a comprehensive understanding of the mechanism of action of probiotics. *Bacillus licheniformis* has been shown to have anti-inflammatory and antioxidant effects and to improve lipid synthesis and beta fatty acid oxidation [37,38]. Similar results were obtained in the present study, where the addition of BL resulted in an enrichment of differential metabolites compared to the control group, mainly in the metabolism of arginine and proline; digestion and absorption of lipids; atherosclerosis; biosynthesis of neomycin, kanamycin, and gentamicin; and regulation of TR channels by inflammatory mediators. *Bacillus licheniformis* has demonstrated the ability to regulate intestinal sub-health by reshaping the intestinal flora, reducing inflammation, and regulating the intestinal flora balance [39]. Additionally, after the addition of SC, the differential metabolites were mainly in the pathways of inflammatory mediators regulating TRP channels, as well as amino acid metabolism, compared to the control group. This addition enhances resistance and immunity against pathogens [40]. The probiotic *Lactobacillus* is generally considered beneficial to both humans and animals, and we found that the addition of *Lactobacillus* affected lipid and amino acid metabolism in *S. trutta*.

The gut microbiota is a complex biological system that plays crucial roles in animals, including the digestion of complex molecules, synthesis of vitamins, and modulation of the immune system [41]. The integration of multiple omics approaches, such as metagenomics and metabolomics, has led to significant advancements in the study of the relationship between gut microbiota and health-related diseases, overcoming the limitations of single-omics studies [42,43]. Previous research has indicated a direct correlation between the differences in gut microbiota composition and significant changes in the metabolomics landscape, including lipid and lipid-related metabolites, amino acids, bile acids, and steroid-related metabolites, after supplementation with probiotics in rainbow trout [44]. In this study, we also observed significant changes in the joint analysis of the microbiota and metabolites in the different probiotic treatment groups, particularly in lipid synthesis and metabolism, organoheterocyclic compounds, and organic acid derivatives. The gut microbiota has a significant effect on lipid synthesis and metabolism. Many probiotics have been shown to provide vitamins, fatty acids, and essential amino acids to the host [7,8,45]. They can also be considered supplementary sources of nutrients [46]. Probiotics improve feed consumption and nutrient absorption, thereby promoting animal growth.

## 5. Conclusions

In conclusion, this study provides evidence that probiotic supplements may improve the growth performance of *S. trutta* by promoting intestinal health. Our findings indicate that probiotic supplementation increases the diversity and richness of the gut microbiota, leading to dynamic changes in microbial composition. Additionally, we investigated the potential mechanisms underlying the interactions between the host and gut microbiome, although the specific molecular regulation mechanism of growth performance changes in S. trutta induced by probiotics needs to be further investigated. Overall, our study highlights the use of multi-omics approaches to comprehensively examine complex host–microbiome interactions, allowing for a more thorough evaluation and exploration of the functional potential of probiotics compared to previous studies. This research contributes to a better understanding of the biological significance and regulatory mechanisms of probiotic supplementation in aquatic animals such as salmon.

## Figures and Tables

**Table 1:** Growth performance of *S. trutta* reared with different probiotics.

Parameters	Group			
	CON	SC	LAB	BL
Initial BW (g)	85.63 ± 2.36	83.63 ± 1.57	86.63 ± 1.23	85.21 ± 2.66
Final BW (g)	158.83 ± 7.68 ^a^	166.83 ± 4.22 ^ab^	173.83 ± 3.65 ^b^	189.83 ± 4.21 ^c^
AWG (%)	85.41 ± 3.86 ^a^	99.47 ± 1.30 ^b^	100.65 ± 1.36 ^b^	122.82 ± 2.02 ^c^
Initial BL (cm)	17.27 ± 0.15	17.22 ± 0.22	17.30 ± 0.16	17.25 ± 0.18
Final BL (cm)	22.45 ± 0.31 ^a^	22.82 ± 0.25 ^a^	23.62 ± 0.28 ^b^	24.01 ± 0.20 ^bc^
AGR (%)	29.99 ± 0.67 ^a^	32.52 ± 0.24 ^b^	36.53 ± 0.36 ^c^	39.19 ± 0.29 ^d^
Survival (%)	92.22 ± 1.92 ^a^	95.56 ± 3.85 ^ab^	97.78 ± 1.92 ^b^	96.67 ± 3.33 ^ab^
Liver body index (%)	1.44 ± 0.01 ^a^	1.43 ± 0.02 ^a^	1.42 ± 0.08 ^ab^	1.40 ± 0.03 ^b^
Fattiness (%)	1.45 ± 0.03 ^a^	1.46 ± 0.02 ^ab^	1.49 ± 0.01 ^ab^	1.52 ± 0.02 ^b^

Note: Values with superscript letters ^a^, ^b^, ^c^, and ^d^ are significantly different across column (*p* < 0.05); ^ab^ indicates no significant difference from ^a^ or ^b^; ^bc^ indicates no significant difference from ^b^ or ^c^. Abbreviations: BW, body weight; BL, body length; AWG, average weight growth; AGR, average growth rate. BL: *Bacillus licheniformis*, SC: *Saccharomyces cerevisiae*, LAB: *lactic acid bacteria*.

## Data Availability

Data will be made available on request.

## Supplementary Materials

The following supporting information can be downloaded at: https://www.mdpi.com/article/10.3390/microorganisms12071410/s1, Table S1: Composition levels of the basal diet (DM basis); Table S2: Nutrient levels of the basal diet (DM basis).

## References

[1] Liu H., Liu Q., Chen Z., Liu Y., Zhou C., Liang Q., Ma C., Zhou J., Pan Y., Chen M. (2018). Draft genome of *Glyptosternon maculatum*, an endemic fish from Tibet Plateau. Gigascience.

[2] Egerton S., Wan A., Murphy K., Collins F., Ahern G., Sugrue I., Busca K., Egan F., Muller N., Whooley J. (2020). Replacing fishmeal with plant protein in Atlantic salmon (*Salmo salar*) diets by supplementation with fish protein hydrolysate. Sci. Rep..

[3] Liu X., Steele J.C., Meng X.-Z. (2017). Usage, residue, and human health risk of antibiotics in Chinese aquaculture: A review. Environ. Pollut..

[4] Monahan C., Nag R., Morris D., Cummins E. (2021). Antibiotic residues in the aquatic environment—Current perspective and risk considerations. J. Environ. Sci. Health Part A-Toxic/Hazard. Subst. Environ. Eng..

[5] Gismondo M.R., Drago L., Lombardi A. (1999). Review of probiotics available to modify gastrointestinal flora. Int. J. Antimicrob. Agents.

[6] De B.C., Meena D.K., Behera B.K., Das P., Das Mohapatra P.K., Sharma A.P. (2014). Probiotics in fish and shellfish culture: Immunomodulatory and ecophysiological responses. Fish Physiol. Biochem..

[7] Balcazar J.L., Vendrell D., de Blas I., Ruiz-Zarzuela I., Girones O., Muzquiz J.L. (2006). Immune modulation by probiotic strains:: Quantification of phagocytosis of Aeromonas salmonicida by leukocytes isolated from gut of rainbow trout (*Oncorhynchus mykiss*) using a radiolabelling assay. Comp. Immunol. Microbiol. Infect. Dis..

[8] Panigrahi A., Azad I.S. (2007). Microbial intervention for better fish health in aquaculture: The Indian scenario. Fish Physiol. Biochem..

[9] Sugita H., Takahashi J., Deguchi Y.J.B. (1992). Production and consumption of biotin by the intestinal microflora of cultured freshwater fishes. Biosci. Biotechnol. Biochem..

[10] Banerjee G., Ray A.K. (2017). The advancement of probiotics research and its application in fish farming industries. Res. Vet. Sci..

[11] Kuebutornye F.K.A., Abarike E.D., Lu Y. (2019). A review on the application of *Bacillus* as probiotics in aquaculture. Fish Shellfish Immunol..

[12] Simon R., Docando F., Nunez-Ortiz N., Tafalla C., Diaz-Rosales P. (2021). Mechanisms Used by Probiotics to Confer Pathogen Resistance to Teleost Fish. Front. Immunol..

[13] Das A., Nakhro K., Chowdhury S., Kamilya D. (2013). Effects of potential probiotic *Bacillus amyloliquifaciens* FPTB16 on systemic and cutaneous mucosal immune responses and disease resistance of catla (*Catla catla*). Fish Shellfish. Immunol..

[14] Kozasa M. (1986). Toyocerin (Bacillus toyoi) as growth promotor for animal feeding. Microbiol. Alim. Nutr..

[15] Vazirzadeh A., Roosta H., Masoumi H., Farhadi A., Jeffs A. (2020). Long-term effects of three probiotics, singular or combined, on serum innate immune parameters and expressions of cytokine genes in rainbow trout during grow-out. Fish Shellfish. Immunol..

[16] Hossain M.K., Islam S.M., Rafiquzzaman S.M., Nuruzzaman M., Hossain M.T., Shahjahan M. (2022). Multi-species probiotics enhance growth of Nile tilapia (*Oreochromis niloticus*) through upgrading gut, liver and muscle health. Aquac. Res..

[17] Li D., Liu C.-M., Luo R., Sadakane K., Lam T.-W. (2015). MEGAHIT: An ultra-fast single-node solution for large and complex metagenomics assembly via succinct de Bruijn graph. Bioinformatics.

[18] Gurevich A., Saveliev V., Vyahhi N., Tesler G. (2013). QUAST: Quality assessment tool for genome assemblies. Bioinformatics.

[19] Zhu W., Lomsadze A., Borodovsky M. (2010). Ab initio gene identification in metagenomic sequences. Nucleic Acids Res..

[20] Zeng D., Chen X., Peng J., Yang C., Peng M., Zhu W., Xie D., He P., Wei P., Lin Y. (2018). Single-molecule long-read sequencing facilitates shrimp transcriptome research. Sci. Rep..

[21] Ashburner M., Ball C.A., Blake J.A., Botstein D., Butler H., Cherry J.M., Davis A.P., Dolinski K., Dwight S.S., Eppig J.T. (2000). Gene ontology: Tool for the unification of biology. The Gene Ontology Consortium. Nat. Genet..

[22] Conesa A., Götz S., García-Gómez J.M., Terol J., Talón M., Robles M. (2005). Blast2GO: A universal tool for annotation, visualization and analysis in functional genomics research. Bioinformatics.

[23] Upadhaya S.D., Rudeaux F., Kim I.H. (2019). Efficacy of dietary Bacillus subtilis and Bacillus licheniformis supplementation continuously in pullet and lay period on egg production, excreta microflora, and egg quality of Hyline-Brown birds. Poult. Sci..

[24] Abdel-Tawwab M., Abdel-Rahman A.M., Ismael N.E.M. (2008). Evaluation of commercial live bakers’ yeast, Saccharomyces cerevisiae as a growth and immunity promoter for Fry *Nile tilapia*, *Oreochromis niloticus* (L.) challenged in situ with *Aeromonas hydrophila*. Aquaculture.

[25] Abu-Elala N., Marzouk M., Moustafa M. (2013). Use of different Saccharomyces cerevisiae biotic forms as immune-modulator and growth promoter for Oreochromis niloticus challenged with some fish pathogens. Int. J. Vet. Sci. Med..

[26] Korni F.M.M., Sleim A.S.A., Abdellatief J.I., Abd-elaziz R.A. (2021). Prevention of vibriosis in sea bass, *Dicentrarchus labrax* using ginger nanoparticles and *Saccharomyces cerevisiae*. J. Fish Pathol..

[27] Li P., Lawrence A.L., Castille F.L., Gatlin D.M. (2007). Preliminary evaluation of a purified nucleotide mixture as a dietary supplement for *Pacific white* shrimp *Litopenaeus vannamei* (Boone). Aquac. Res..

[28] Wache Y., Auffray F., Gatesoupe F.-J., Zambonino J., Gayet V., Labbe L., Quentel C. (2006). Cross effects of the strain of dietary Saccharomyces cerevisiae and rearing conditions on the onset of intestinal microbiota and digestive enzymes in rainbow trout, Onchorhynchus mykiss, fry. Aquaculture.

[29] Fuller R. (1989). Probiotics in man and animals. J. Appl. Bacteriol..

[30] Saarela M., Hallamaa K., Mattila-Sandholm T., Mättö J. (2003). The effect of lactose derivatives lactulose, lactitol and lactobionic acid on the functional and technological properties of potentially probiotic Lactobacillus strains. Int. Dairy J..

[31] Kuebutornye F.K.A., Abarike E.D., Lu Y., Hlordzi V., Sakyi M.E., Afriyie G., Wang Z., Li Y., Xie C.X. (2020). Mechanisms and the role of probiotic Bacillus in mitigating fish pathogens in aquaculture. Fish Physiol. Biochem..

[32] Qin L., Xiang J., Xiong F., Wang G., Zou H., Li W., Li M., Wu S. (2020). Effects of *Bacillus licheniformis* on the growth, antioxidant capacity, intestinal barrier and disease resistance of grass carp (*Ctenopharyngodon idella*). Fish Shellfish Immunol..

[33] He Y., Zhang K., Li S., Lu X., Zhao H., Guan C., Huang X., Shi Y., Kang Z., Fan Y. (2023). Multiomics analysis reveals the molecular mechanisms underlying virulence in Rhizoctonia and jasmonic acid-mediated resistance in *Tartary buckwheat* (*Fagopyrum tataricum*). Plant Cell.

[34] Stentiford G.D., Feist S.W., Stone D.M., Bateman K.S., Dunn A.M. (2013). Microsporidia: Diverse, dynamic, and emergent pathogens in aquatic systems. Trends Parasitol..

[35] Ding Z., Pan J., Huang H., Jiang G., Chen J., Zhu X., Wang R., Xu G. (2018). An integrated metabolic consequence of Hepatospora eriocheir infection in the Chinese mitten crab Eriocheir sinensis. Fish Shellfish. Immunol..

[36] Agus A., Clement K., Sokol H. (2021). Gut microbiota-derived metabolites as central regulators in metabolic disorders. Gut.

[37] Chen S., Ye W., Clements K.D., Zan Z., Zhao W., Zou H., Wang G., Wu S. (2023). *Bacillus licheniformis* FA6 Affects Zebrafish Lipid Metabolism through Promoting Acetyl-CoA Synthesis and Inhibiting β-Oxidation. Int. J. Mol. Sci..

[38] Pezsa N.P., Kovacs D., Racz B., Farkas O. (2022). Effects of Bacillus licheniformis and Bacillus subtilis on Gut Barrier Function, Proinflammatory Response, ROS Production and Pathogen Inhibition Properties in IPEC-J2-Escherichia coliSalmonella Typhimurium Co-Culture. Microorganisms.

[39] Feng S., Meng C., Hao Z., Liu H. (2022). *Bacillus licheniformis* Reshapes the Gut Microbiota to Alleviate the Subhealth. Nutrients.

[40] Chiu C.-H., Cheng C.-H., Gua W.-R., Guu Y.-K., Cheng W. (2010). Dietary administration of the probiotic, *Saccharomyces cerevisiae* P13, enhanced the growth, innate immune responses, and disease resistance of the grouper, *Epinephelus coioides*. Fish Shellfish. Immunol..

[41] Zhang L., Chen F., Zeng Z., Xu M., Sun F., Yang L., Bi X., Lin Y., Gao Y., Hao H. (2021). Advances in Metagenomics and Its Application in Environmental Microorganisms. Front. Microbiol..

[42] Liu R., Hong J., Xu X., Feng Q., Zhang D., Gu Y., Shi J., Zhao S., Liu W., Wang X. (2017). Gut microbiome and serum metabolome alterations in obesity and after weight-loss intervention. Nat. Med..

[43] Patti G.J., Yanes O., Siuzdak G. (2012). Metabolomics: The apogee of the omics trilogy. Nat. Rev. Mol. Cell Biol..

[44] Rasmussen J.A., Villumsen K.R., Ernst M., Hansen M., Forberg T., Gopalakrishnan S., Gilbert M.T.P., Bojesen A.M., Kristiansen K., Limborg M.T. (2022). A multi-omics approach unravels metagenomic and metabolic alterations of a probiotic and synbiotic additive in rainbow trout (*Oncorhynchus mykiss*). Microbiome.

[45] Sugita H., Miyajima C., Deguchi Y.J.A. (1991). The vitamin B12-producing ability of the intestinal microflora of freshwater fish. Aquaculture.

[46] Verschuere L., Rombaut G., Sorgeloos P., Verstraete W.J.M. (2000). Probiotic bacteria as biological control agents in aquaculture. Microbiol. Mol. Biol. Rev..

