# Supplementary material for: Multi-Omics Analysis Reveals the Regulatory Mechanism of Different Probiotics on Growth Performance and Intestinal Health of Salmo trutta (S. trutta)
Source: Microorganisms. 2024 Jul 12;12(7):1410. doi: 10.3390/microorganisms12071410 (PMC11278557; doi:10.3390/microorganisms12071410)
Supplement: Supplementary file 1 [file microorganisms-12-01410-s001.zip › microorganisms-3108956-supplementary.pdf]

Supplementary material

TABLE S1. Composition levels of the basal diet（DM basis）

**Ingredients**	**Diets /%**
Wheat middings	11.47
Wheat flour	10
Sonybean concentrated protein	22.93
Fish meal	35
Fish oil	7.35
Soybean oil	7.35
Cellulose	0.43
$Ca(H_2PO_4)_2$	0.89
Squid liver paste	1
Soybean lecithin	2
Ethoxy quin	0.05
Premix[1)]	1
Met	0.53
Total	100

Note:[1)]indicates the premix provided the following per kg of diets: VC 1000 mg, VE 60 mg, VK3 5 mg , VA 15000 IU, VD3 3000 IU, VB1 15 mg,VB2 30 mg, VB6 15 mg, VB12 0.5 mg, 175 mg, nicotinic acid 5 mg/kg, inositol 300 mg, biotin 2.5 mg, pantothenic acid 50 mg, choline 2 g, antimildew 200 mg, Mg 1 g, Fe 25 mg, Cu 3 mg, Mn 15 mg, I 0.6 mg.

Supplementary material

TABLE S2. Nutrient levels of the basal diet（DM basis）

Nutrient levels[2)]	Diets /%
GE/（MJ/kg）	20.71
CP	45
EE	18

Note:[2)] indicates measured values.
